# Medical Information Provided by Transgender and Gender-Diverse Content Creators on YouTube: Descriptive Content Analysis

**DOI:** 10.2196/75787

**Published:** 2025-08-29

**Authors:** Lydia Bliss, Qianqian Zhao, Irene Chao, Oliver L Haimson, Ellen Selkie

**Affiliations:** 1 Department of Pediatrics, School of Medicine and Public Health, University of Wisconsin–Madison, 800 University Bay Drive, Office 300-21, Madison, WI, 53705, United States, 1 6084454231; 2 Department of Biostatistics and Medical Informatics, School of Medicine and Public Health, University of Wisconsin–Madison, Madison, WI, United States; 3 School of Information, University of Michigan, Ann Arbor, MI, United States

**Keywords:** transgender, transgender youth, gender-affirming care, YouTube, social media, content creators, influencers, social media health information

## Abstract

**Background:**

Transgender and gender-diverse (TGD) individuals frequently turn to social media to find community, express their identities, and access essential information. These platforms are easily accessible to TGD people and enable health information–seeking in anonymous, identity-affirming spaces outside of traditional health care systems. As a result, social media has become a critical source of health information on topics like gender-affirming care for TGD individuals, specifically for TGD youth. YouTube, one of the most widely used social media platforms, is especially popular for its long-form videos made by content creators who have built dedicated followings on the platform. Among them are TGD content creators, many of whom make content documenting their medical transition and gender identity journey and provide general information about TGD topics. TGD creator content therefore makes YouTube an important platform for health education for TGD individuals.

**Objective:**

This study aims to describe the health-related content shared by TGD content creators on YouTube. Specifically, we characterize the medical topics addressed, the frameworks used to discuss these topics, and the valence of creators’ health care experiences.

**Methods:**

A descriptive content analysis was performed on 2485 videos posted by 42 self-identified TGD YouTube content creators. Videos were systematically evaluated for mentions of gender-affirming care and other health-related topics. We also examined whether creators framed medical information using personal narratives or an informational approach and if they characterized their medical experiences as positive, negative, or neutral.

**Results:**

Most videos (n=1724, 69.4%) created by TGD content creators did not include discussions related to gender identity or transitioning. However, among the videos that did address gender identity (n=761, 30.6%), mentions of medical topics were prevalent (n=554, 72.8%). Of videos that discussed medical topics, gender-affirming surgeries (n=356, 64.3%) and hormone replacement therapy (n=307, 55.4%) were the most frequently discussed. Other commonly discussed medical topics included mental health (n=131, 23.6%) and sexual health (n=96, 17.3%). Videos covering medical topics primarily centered on personal experiences (n=411, 74.2%), with content creators often characterizing these experiences positively (n=224, 73.2%).

**Conclusions:**

This study highlights the breadth of health-related information shared by TGD content creators on YouTube. Our findings underscore the role of long-form video content on YouTube as an educational resource for TGD people, offering health information that is both easy to access and grounded in lived experience. Clinicians can use these findings to better understand the health information that their TGD clients are likely to encounter online, fostering more informed and supportive conversations about gender-affirming care.

## Introduction

Transgender and gender-diverse (TGD) individuals have been known to use social media to fulfill a variety of social, emotional, and informational needs. TGD individuals use social media as a vital space for seeking support, connecting with others, and developing their identity, often curating their online environments for these purposes [1-6]. These behaviors are especially significant for TGD youth, who use the internet and social media more than their cisgender peers, particularly as a resource for exploring their gender identity [7-10]. Importantly, online spaces improve information accessibility, enabling individuals to seek advice about gender identity that may be difficult to obtain through traditional means or uncomfortable to discuss with offline support networks [11,12].

Given the significant barriers that TGD individuals often encounter in accessing medical care, social media plays a critical role in providing a place to address health-related questions, specifically about gender-affirming care [10]. Seeking health information online has become a preferred method for many TGD individuals due to its accessibility, convenience, affordability, and the option for anonymity [12-14]. Social media provides safe spaces for community members to share information while offering diverse formats for consuming it, such as real-time answers in online forums or archived content on platforms like YouTube [13]. Furthermore, TGD individuals have highlighted the value of social media for accessing firsthand accounts of those who have undergone gender-affirming treatments [13].

Seeking health-related information online is particularly common among TGD youth. Herrmann et al [4] found that all TGD adolescent participants used social media or the internet to seek information on transgender-related topics, most commonly gender-affirming medical interventions. Selkie et al [15] reported similar findings, with TGD adolescents identifying informational support as a helpful aspect of social media, specifically for topics like gender-affirming care. Importantly, in both studies, YouTube was identified as the most common web-based platform for TGD adolescents to seek transgender-specific content [4,15].

YouTube, a global video-sharing platform, is the most commonly used social media platform among Americans, used by 83% of adults and 93% of teens [16,17]. As the second-most visited site in the world (behind only Google), YouTube has proven to be a dominant and enduring platform in the social media landscape [18]. Compared to other social media platforms, YouTube is uniquely suited to serve as an educational resource. YouTube videos tend to be significantly longer than videos shared on other platforms and can range from a few minutes to over an hour. These long-form videos allow for the sharing of more detailed and in-depth videos where creators can share their experiences, opinions, and knowledge through a variety of genres, including video blogs, story times, and commentary. This extended format supports the sharing of health-related content through a variety of informative styles, often centered on sharing personal experiences [19].

Much of the popular content available on YouTube is made by those who have gained a substantial online following. In this paper, they are referred to as “content creators” or simply “creators,” although they are also commonly known as “YouTubers” or “influencers.” Content creators build a following through their self-branded content and are important figures online: they are drivers of internet discourse, create spaces for like-minded viewers, and disseminate content that is seen as relatable to their audience. As such, content creators are known to cultivate strong fan communities, frequently made up of younger viewers [20].

A content creator’s ability to remain both authentic and personable, while simultaneously maintaining the reputation of that of a celebrity figure, serves to amplify their influence among their audience [21]. Familiar content creators have been shown to be perceived as more trustworthy and similar to oneself than traditional celebrities, showing that they may be particularly able to influence behaviors among audiences seeking validation and guidance [22]. Because these creators are often regarded as important role models to their viewers, they are uniquely positioned to serve as influential health educators [23]. Bandura’s [24] Social Cognitive Theory of Mass Communication posits that individuals learn by observing media figures and that health media messaging is most effective when it promotes self-efficacy—the belief in one’s ability to exercise control over their own health. As personable and relatable figures, content creators may promote a sense of empowerment in viewers, influencing their health behaviors.

TGD content creators have a considerable presence on YouTube and are known to make content about gender-affirming care. A popular format for sharing this information is the transition vlog—a video blog where creators document their physical and emotional changes resulting from gender-affirming care interventions such as hormone replacement therapy or surgery [25]. This content has been shown to be valuable to TGD viewers, who watch these transition vlogs to learn about transition processes and experiences [26].

Although a number of scholars have examined TGD content and experiences on YouTube, only one study to date has systematically analyzed the content made by popular TGD content creators [27-38]. In an analysis of the content made by 8 TGD creators, Miller [38] found that 53.5% of their videos provided educational insights on transgender topics, with 34.2% of videos discussing elements of physical transition, most commonly hormones or surgery. However, the breadth and frequency of medical topics covered by TGD content creators remains unexplored at a large scale. Further research is needed to assess the health-related information shared by these creators, given the diversity of TGD contributors and the vast range of content on the platform. It is also important to examine how this information is framed and how creators portray their medical experiences as this may shape how viewers interpret and respond to their content.

Although online content is recognized as an important resource for TGD individuals, limited research has examined the actual content provided. Content analysis is a commonly used method in social media research to characterize and describe health-related information online [39]. It is especially useful for examining the rhetoric, topics, and emerging themes in online content [10,38,40,41]. This approach allows for the direct observation of material that social media users encounter; as it does not rely on participants’ memories or interpretations, content analysis offers a more accurate and unobtrusive method for evaluating online health information.

Given YouTube’s popularity and the influence of its creators, understanding the platform’s medical content is essential. This exploratory study aims to describe the health-related content shared by TGD content creators on YouTube by analyzing the medical topics they address, the frameworks they use to discuss these topics, and the valence of their health care experiences.

## Methods

### Study Design

A descriptive content analysis study was conducted between June 2021 and October 2024. This analysis was part of a larger study evaluating the videos posted by TGD content creators discussing gender identity and transitioning. This analysis focused specifically on videos discussing medical topics.

### Content Creator Identification

Recommendations for TGD content creators were first obtained from a large convenience sample of TGD young people, researchers, and health professionals serving TGD individuals in June 2021. This approach prioritized community relevance in the selection of creators and avoided reliance on YouTube’s recommendations, which risked introducing algorithmic bias. Recommended content creators were individually evaluated for their internet presence and following across YouTube, Instagram, Twitter (now X), and/or TikTok. Accounts on each platform were evaluated against 4 criteria: content in English, identification as TGD by the creator, at least 10,000 followers, and a minimum of 10 posts within the two years prior to data collection. Content creators with a YouTube channel and at least 1 social media account on any platform that met all 4 criteria were selected for further investigation. We included creators with large followings on other social media platforms, even if they did not meet the follower threshold on their specific YouTube channels. This preserved our intent to examine long-form video content while acknowledging that popular influencers do not limit their presence to one platform. Moreover, research indicates that even content creators with relatively small followings (commonly referred to as micro-influencers) can still have a substantial influence over their audience, comparable to those with larger followings [42,43].

The research team was comprised of academic researchers and gender-affirming care clinicians representing diverse gender identities, including lived TGD experience. The team met regularly to evaluate the initial group of YouTube accounts meeting the inclusion criteria. The accounts were reviewed for post history, content type, and the overarching narrative of the channel. The research team reviewed each account to reach consensus on whether to include or exclude the creator from the final analysis. Since our objective was to examine content specific to gender identity and transitioning, creators were determined as fit for inclusion if they posted regularly about these topics. Excluded creators focused on other topics within the LGBTQ+ community (such as advocacy), posted about other topics entirely (such as music), or posted minimally in recent years. This process yielded a total of 42 eligible YouTube content creators.

### Ethical Considerations

Because the data were publicly accessible, this study did not constitute human subjects research and did not require review by the University of Wisconsin–Madison Institutional Review Board [44,45]. However, analyzing public social media data raises important ethical considerations. Although content posted to social media may be technically public, Buck and Ralston [46] argue that this does not automatically make online content “public data” in a research context. They note that social media users hold different expectations of privacy depending on how or where they share their content. Accordingly, privacy should instead be understood in terms of the user’s intended audience and their expected reach, rather than their content’s technical availability [46]. Such expectations vary significantly among different users; while average social media users may reasonably expect their posts to remain within their personal networks, content creators typically create videos with the specific intention of reaching a broader audience, suggesting a lower expectation of privacy.

Still, while content creators often aim to reach larger audiences, they may reasonably expect their content to stay within TGD communities or among supportive viewers. When amplified beyond these circles, they can become targets of significant online harassment, especially within online spaces that have been known to be hostile toward the TGD community [47-49]. Publicly identifying a list of individual TGD creators in this paper could increase risk for targeted harassment; therefore, anonymizing their identities is essential to minimizing potential harm.

To protect creators’ privacy, we took several precautions. First, we avoided using automated scraping tools for data collection, instead using human coders to manually collect metadata and analyze video content. Second, all identifiable information, such as creator names, channel names, and video titles, were deidentified for reporting. Example quotes reported in this paper are paraphrases rather than direct quotes from creators. Lastly, our analysis focused on characterizing the types of content present across the full body of videos, rather than on the individual creators themselves.

### Codebook Development

A team-based, iterative approach was used to develop a structured codebook for content analysis, consistent with prior content analyses [50-52]. As to not interfere with the coding of chosen accounts, a sample of videos were pulled from the channels of creators that were not selected for final analysis but whose content closely resembled the content made by the included creators. The core research team evaluated these posts to identify variables related to gender identity development and transitioning in both medical and nonmedical contexts. Once the preliminary codebook was created, members of the research team independently applied the codebook to an additional set of nonstudy posts. The research team regularly convened to review coding agreements and disagreements, refining the codebook with emergent themes and clarifying existing codes until reaching consensus. The full, expanded codebook (including nonmedical topics not included in this analysis) is available in Multimedia Appendix 1.

### Coder Training and Agreement

Five coders were trained by evaluating 20 nonstudy posts and comparing their coding to that of a lead researcher [50]. Coding of study data began after each of the 5 coders reached 80% agreement or higher with the lead researcher across all codes and posts. All coding disagreements were discussed to refine coder interpretation and ensure accurate application of the codebook.

During active periods of study coding, paired indexing and weekly team meetings were conducted to periodically assess interrater agreement and resolve questionable codes by consensus [53-55]. Any necessary refinements to the codebook were discussed and implemented. During data collection, interrater agreement was assessed between the 2 primary coders who analyzed the majority of the dataset, coding 534 of 761 (70.2%) of the included videos. Agreement was calculated based on a subsample of 10 jointly coded videos. Results indicated strong reliability: prevalence-adjusted and bias-adjusted κ=0.93 (SE=0.0087), 95% (CI 0.9118-0.9460); Gwet AC1=0.96 (SE=0.0051), 95% CI (0.9497-0.9697). Multimedia Appendix 2 presents the percent observed agreement, prevalence-adjusted and bias-adjusted κ, and Gwet AC1 for each code.

### Content Coding

#### Overview

Coding of YouTube videos was conducted from January 2023 to February 2024. Coders reviewed one channel at a time from the 42 YouTube content creators selected for analysis. Each channel was reviewed by 1 coder, which ensured continuity and context in coding this large dataset. Videos from each channel were reviewed in order of popularity, using YouTube’s sorting feature to organize the videos by view count. To ensure theoretical saturation, coders reviewed every video on the account until 20 videos that met inclusion criteria were coded. To meet inclusion criteria, a video had to explicitly mention topics related to gender identity or the medical, social, or legal aspects of transitioning. The number of likes and views were collected for videos meeting inclusion criteria. The number of subscribers of each channel was recorded after the content coding of all channels was complete.

Videos were analyzed based on the creators’ personal mentions of their gender identity, the medical topics that they addressed, the frameworks used to discuss medical topics, and the valence of their medical experiences. Coders used Qualtrics to record their evaluations of each video. Codes were marked as present or not present; for codes marked as present, coders explained their decisions in a corresponding textbox.

#### Medical Topics

Discussions of medical topics were categorized into subtopics: puberty and androgen blockers, hormone replacement therapy, menstruation, surgery, mental health, sexual health, fertility and family planning, treatment regrets, and general mentions of medical topics (Table 1). Medical topics were coded if they were discussed in the video at least once.

**Table 1.** Codebook of medical topics identified in YouTube videos made by selected transgender and gender-diverse content creators.

Code	Definition	Subcodes^a^
Puberty and androgen blockers	Discussions about medications to delay puberty or block androgens. Includes personal experience and/or general information.	N/A^b^
Hormone replacement therapy	Discussions about hormone replacement therapy. Includes personal experience and/or general information.	Estrogen, testosterone, not specified
Surgery	Discussions about surgical interventions for gender-affirming care. Includes personal experience and/or general information.	Top surgery (gender-affirming chest surgery), bottom surgery (gender-affirming genital surgery), facial feminization surgery, Brazilian butt lift, general mentions, other
Mental health	Discussions about mental health diagnoses, treatments, and therapies. Includes personal experience and/or general information.	Medication, treatment, and therapy; therapists/psychologists; reparative/conversion therapy; suicidality; eating disorders; substance use disorder/addiction; posttraumatic stress disorder; other diagnoses; general mentions
Menstruation	Discussions about the management of periods and symptoms associated with menstruation. Includes personal experience and/or general information.	N/A
Sexual health	Discussions about safe sex practices, sexual boundaries, sexual function, sexually transmitted infections, and consent. Includes personal experience and/or general information.	N/A
Fertility and family planning	Discussions about the ability to conceive or have biological children or alternative plans for creating a family, such as adoption or surrogacy. Includes personal experience and/or general information.	N/A
Treatment regrets	Expressing regret about undergoing a certain treatment or regretting a certain aspect of that treatment. Includes personal experience and/or general information.	N/A
General mentions	General mentions of medically transitioning and gender-affirming health care, without naming a specific treatment. Includes personal experience and/or general information.	N/A

a Codes were marked as present or not present.

b N/A: not applicable.

#### Medical Discussion Frameworks

All discussions about medical topics were categorized by framework. Discussions were categorized as “personal” when the creator’s statements were self-referential, usually by sharing personal experiences, feelings, and opinions. Discussions that presented facts, advice, or explanations were categorized as “informational.” Discussions that could not be categorized as personal or informational were categorized as “other” (Table 2).

**Table 2.** Codebook of the framework and valence of medical topics discussed by selected transgender and gender-diverse YouTube content creators.

Framework	Definition	Valence (if applicable)^a^
Personal	Statements are self-referential. Creator shares personal experiences, feelings, and opinions.	Positive (affirming experiences), negative (disaffirming experiences), or neutral/not applicable (neither positive nor negative)
Informational	Statements are not self-referential. Creator presents information as facts, explanations, or advice.	N/A^b^
Other	Statements are neither personal nor informational.	N/A

a Valence assessed for creators’ personal discussions of surgical and pharmacological interventions directly related to gender-affirming care.

b N/A: not applicable.

#### Valence of Medical Experiences

**Figure 1.**
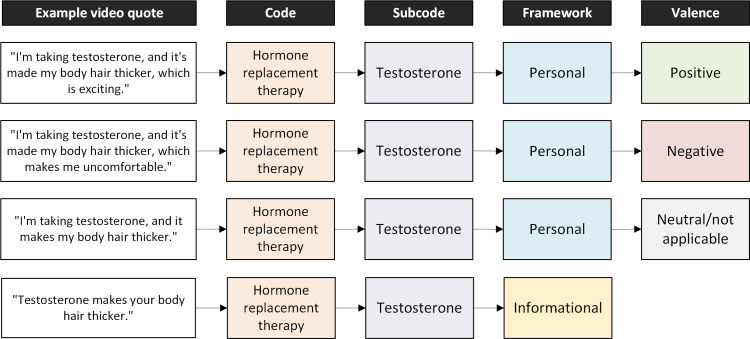
Example quotes from transgender and gender-diverse content creator videos with corresponding codes.

Valence was assigned to creators’ personal discussions of surgical and pharmacological interventions directly related to gender-affirming care. Valence refers to the creators’ characterization of their medical experiences, with positive indicating desirable or affirming experiences, and negative indicating undesirable or disaffirming experiences. Experiences were categorized as being positive, negative, or neutral/not applicable (Table 2). Figure 1 illustrates example quotes coded according to our codebook.

## Results

**Figure 2.**
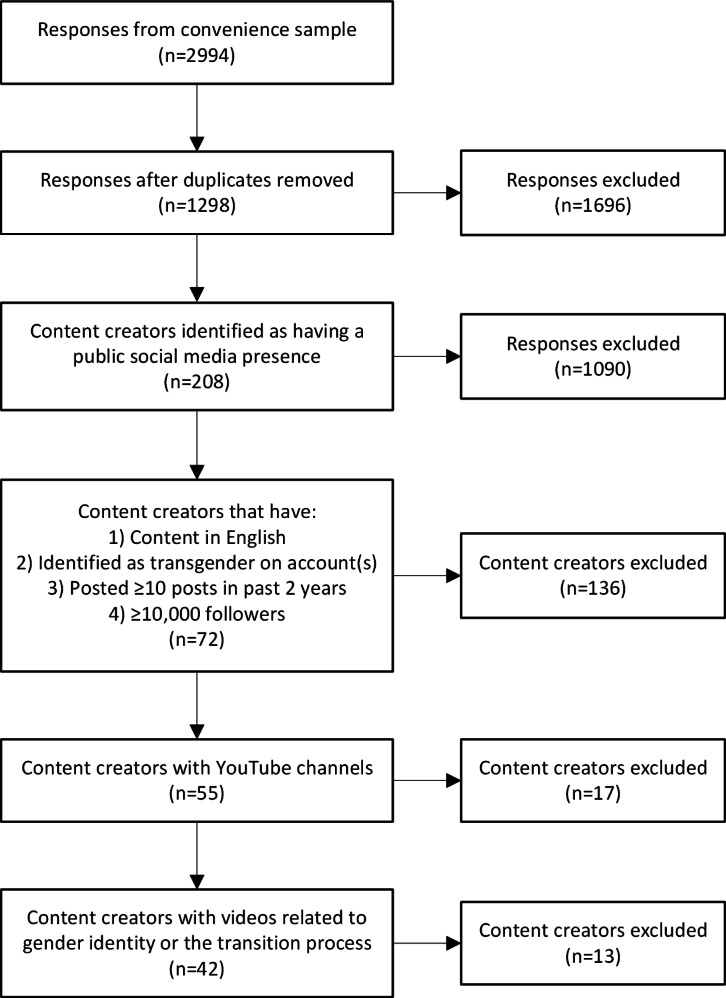
Transgender and gender-diverse content creator selection process.

A total of 42 content creators were included in the analysis. Figure 2 presents a flowchart of the creator selection process.

### Creator Gender and Channel Engagement

#### Gender Identity

Gender identity labels that creators used to describe themselves in more than 1 video were used in analysis. Gender identities reported are not mutually exclusive, to reflect creators who identified with multiple genders. Creators described their personal gender identities as 45.2% transgender male (n=19), 21.4% transgender female (n=9), 23.8% transgender (n=10), 21.4% nonbinary (n=9), 7.1% gender queer (n=3), and 9.5% additional gender identities (n=4). In addition, 9.5% of creators did not specify their gender identities more than once (n=4).

#### Subscribers

The number of subscribers to the creators’ YouTube channels varied widely, ranging from 160 to 3,410,000. The mean number of subscribers was 492,976 (SD 799,317), while the median was 117,000 (IQR 618,800).

#### Video Engagement

The average number of likes and views for all videos in the sample (n=761) was 24,997 (SD 42,192) and 702,961 (SD 2,303,544), respectively. Given the skewed distribution of engagement metrics, medians and IQRs are also reported (Tables 3-4). Patterns were largely consistent when comparing videos with and without discussions of medical topics, with medical topic videos showing slightly higher average engagement but similar medians. A full comparison of likes and views for all videos, medical-topic videos, and nonmedical videos is available in Tables 3-4.

**Table 3.** Likes across all videos, medical-topic videos, and nonmedical videos made by selected transgender and gender-diverse YouTube content creators.

Video type	Mean (SD)	Median (IQR)
All videos in sample (n=761)	24,997 (42,192)	8650 (29,400)
Nonmedical (n=207)	24,521 (31,706)	9300 (40,700)
Medical (n=554)	25,176 (45,518)	8600 (25,700)

**Table 4.** Views across all videos, medical-topic videos, and nonmedical videos made by selected transgender and gender-diverse YouTube content creators.

Video type	Mean (SD)	Median (IQR)
All videos in sample (n=761)	702,961 (2,303,544)	221,711 (702,974)
Nonmedical (n=207)	560,002 (784,340)	237,785 (768,292)
Medical (n=554)	756,377 (2,655,731)	216,342 (703,509)

### Medical Topics

Of the videos analyzed (n=2485), 30.6% (n=761) of videos included discussions directly related to gender identity or transitioning. Of these videos, 72.8% (n=554) discussed medical topics at least once. Videos that did not discuss medical topics addressed other topics related to gender identity and the transition process, including nonmedical affirming activities (eg, clothing, makeup), social experiences, and legal sex and name changes (Multimedia Appendix 1). Further analysis included videos that discussed a medical topic at least once.

The most frequently discussed medical topics were surgery (n=356, 64.3%) and hormone replacement therapy (n=307, 55.4%; Table 5). Testosterone was the most frequently discussed type of hormone replacement therapy (n=205, 37%), while top surgery was the most frequently discussed type of surgical intervention (n=193, 34.8%). Other commonly discussed medical topics include mental health (n=131, 23.6%) and sexual health (n=96, 17.3%).

**Table 5.** Proportion of videos made by selected transgender and gender-diverse YouTube content creators regarding specific medical topics (N=554).

Code and subcode		Number of videos^a^	Percent of videos^b^
Puberty and androgen blockers		48	8.7
Hormone replacement therapy		307	55.4
	Estrogen	31	5.6
	Testosterone	205	37
	Not specified	92	16.6
Menstruation		24	4.3
Surgery		356	64.3
	Top surgery (chest surgery)	193	34.8
	Bottom surgery (genital surgery)	111	20
	Facial feminization surgery	34	6.1
	Brazilian butt lift	7	1.3
	General mentions	116	20.9
	Other	13	2.3
Mental health		131	23.6
	Medication, treatment, and therapy	36	6.5
	Therapists/psychologists	5	0.9
	Reparative/conversion therapy	10	1.8
	Suicidality	29	5.2
	Eating disorders	3	0.5
	Substance use disorder/addiction	5	0.9
	Depression	40	7.2
	Anxiety	21	3.8
	Other diagnoses	5	0.9
	General mentions	33	6
Sexual health		96	17.3
Fertility and family planning		36	6.5
Treatment regrets		9	1.6
General mentions		69	12.5

a Medical topics were not mutually exclusive. Videos discussing more than one topic are included in each relevant category. Videos that discussed a medical topic at least once were counted.

b Compared to all videos discussing medical topics (N=554).

### Medical Discussion Frameworks

Of all videos where creators discussed medical topics (n=554), 74.2% of videos (n=411) included discussions categorized as personal, 55.8% of videos (n=309) included discussions categorized as informational, and 70.9% of videos (n=393) included discussions categorized as other. Medical frameworks were not mutually exclusive. Videos using more than one framework were included in each relevant category.

### Valence of Medical Experiences

Of all videos where creators shared their personal experiences with gender-affirming care (n=306), 73.2% of videos (n=224) discussed positive experiences, 33.7% (n=103) discussed negative experiences, and 61.1% (n=187) discussed experiences that were neutral or not applicable. Positive, negative, and neutral categories were not mutually exclusive, since videos could include discussions of multiple experiences with different valences. Videos that discussed medical experiences with different valences were included in each relevant category.

## Discussion

### Relevance

Given the unique health needs and challenges faced by TGD individuals, access to health information is essential to support their well-being and empower them to make informed decisions about their care. However, TGD individuals experience significant barriers to accessing health services and are less likely to use both primary and specialty care services compared to cisgender individuals [56-58]. These challenges are even greater for the estimated 113,900 transgender youth (over one-third of transgender youth in the United States) who live in a state that has enacted a ban on gender-affirming care [59]. Additionally, an executive order issued by the Trump administration that aims to end federal funding for health care organizations providing gender-affirming care reflects a broader effort to restrict access to such care for transgender youth in the United States [60]. Without accessible systems in place, TGD individuals may turn to alternative sources for health information, such as social media. It is therefore important to understand the medical content available on popular platforms such as YouTube, which is widely used among TGD youth [4,15].

### Principal Results

This study described the health-related content made by TGD content creators on YouTube. This study found that most content created by transgender creators did not center on transgender-specific topics. Instead, these creators showcased multiple aspects of their lives beyond their gender identity across their videos. This variety of content may allow TGD followers to connect with creators over additional shared interests, fostering a stronger sense of kinship with the creator, enhancing their relatability and influence.

When TGD content creators discussed their gender identity, medical topics frequently emerged. These findings indicate that creators commonly highlight the role of medical care in affirming their gender identity. This suggests that TGD individuals engaging with such content, particularly for gender identity development purposes, are likely to encounter information about gender-affirming health care.

Surgery and hormone replacement therapy were common topics, with testosterone and top surgery discussed most frequently. Other commonly discussed medical topics included mental health and sexual health. This range of topics highlights a holistic perspective of gender-affirming care, with discussions extending beyond physical interventions to include broader aspects of health.

Most videos including discussions of medical topics focused on personal experiences, aligning with previous research that highlights social media’s role in sharing firsthand medical narratives [13,19]. Compared to the smaller sample sizes of previous studies, our findings indicate that this trend is evident across the wider genre of TGD medical content. Such videos offer insights grounded in lived experience, providing information from those who have undergone gender-affirming treatments, which may be especially helpful for informing viewers’ medical decisions. Notably, the content often reflected positive experiences, suggesting that content creators tend to report or emphasize favorable outcomes from gender-affirming care.

### Implications

Although it is known that TGD YouTubers discuss medical topics, prior studies have largely relied on small samples. To our knowledge, no study has systematically categorized this content at scale. Our study contributes new insights by using a larger dataset and a more granular, structured codebook to identify the frequency and framing of medical discussions across a wide range of videos.

These findings may be especially insightful for clinicians working with TGD individuals. Clinicians are likely aware that their clients are viewing online videos related to their experiences. Providers may find it useful to ask clients about specific stories they may have seen on social media and use their responses to build a discussion about individual goals and expectations for treatment. By understanding the types of narratives their clients are exposed to, clinicians can better contextualize their clients’ perspectives and tailor their guidance accordingly.

### Limitations

The large number of YouTube videos included in this study enhances its generalizability and depth of findings across a diverse range of content creators. However, while the study expands on our understanding of creators on YouTube, this focus also excludes insights from emerging creators present on other platforms like Instagram and TikTok. Furthermore, this study did not include short-form content (ie, YouTube Shorts), which has risen in popularity in recent years. Possible differences between long-form and short-form health content across social media platforms should be examined in subsequent studies.

This study also used a convenience sample to identify content creators, aiming to reflect the viewing habits of TGD individuals, rather than relying on algorithm-driven recommendations on the YouTube platform. Although this strategy helped prioritize community relevance in our sampling, it also introduces selection bias. We also limited our sample to English-speaking creators, which limits cultural context for non-English speaking TGD individuals. Additionally, our analysis of channel subscriber and engagement metrics (ie, likes and views) did not account for time-dependent factors, such as how long individual videos have been online or the age of the channel. Future analyses may account for these variables to better contextualize engagement patterns over time.

This study also did not assess the accuracy of health information made by content creators. Although these content creators have lived experience with gender-affirming medical treatments, they are not medical professionals, and their content is not held to clinical standards. As a result, there is the potential for this content to contain inaccurate or misleading information. Assessing the accuracy of health information shared by TGD content creators is a vital area for future study given broader concerns about medical misinformation online.

### Conclusions

This study offers insights into the health-related information shared by TGD content creators on YouTube, emphasizing the range of topics covered and the approaches used to share medical information. Although most videos by TGD content creators are unrelated to gender identity, videos that do address this topic frequently mention gender-affirming medical care. Our findings underscore the potential of social media sites like YouTube to serve as a vital resource for TGD individuals seeking accessible and relatable health information, particularly in the face of systemic barriers to accessing inclusive health care.

## Supplementary material

10.2196/75787 Multimedia Appendix 1 Full codebook of medical and nonmedical topics identified in YouTube videos made by selected transgender and gender-diverse content creators.

10.2196/75787 Multimedia Appendix 2 Summary of interrater reliability statistics.

## Abbreviations

TGD: transgender and gender-diverse
