# Supplementary material for: Medical Information Provided by Transgender and Gender-Diverse Content Creators on YouTube: Descriptive Content Analysis
Source: JMIR Form Res. 2025 Aug 29;9:e75787. doi: 10.2196/75787 (PMC12396793; doi:10.2196/75787)
Supplement: Multimedia Appendix 1 [file formative-v9-e75787-s001.docx]

**Appendix 1. Full codebook of medical and non-medical topics identified in YouTube videos made by selected TGD content creators.**

| Medical Codes | | | | |
| --- | --- | --- | --- | --- |
| **Code** | **Definition** | **Subcode** | Framework | Valence |
| **Puberty and Androgen Blockers** | Discussions about medications to delay puberty or block androgens. Includes personal experience and/or general information. |  | Personal | Positive |
|  |  |  |  | Negative |
|  |  |  |  | Neutral |
|  |  |  | Informational | |
|  |  |  | Other | |
| **Hormone Replacement Therapy** | Discussions about hormone replacement therapy (HRT). Includes personal experience and/or general information. | Estrogen | Personal | Positive |
|  |  |  |  | Negative |
|  |  |  |  | Neutral |
|  |  |  | Informational | |
|  |  |  | Other | |
|  |  | Testosterone | Personal | Positive |
|  |  |  |  | Negative |
|  |  |  |  | Neutral |
|  |  |  | Informational | |
|  |  |  | Other | |
|  |  | Not specified | Personal | Positive |
|  |  |  |  | Negative |
|  |  |  |  | Neutral |
|  |  |  | Informational | |
|  |  |  | Other | |
| **Surgery** | Discussions about surgical interventions for gender-affirming care. Includes personal experience and/or general information. | Top Surgery  (gender-affirming chest surgery) | Personal | Positive |
|  |  |  |  | Negative |
|  |  |  |  | Neutral |
|  |  |  | Informational | |
|  |  |  | Other | |
|  |  | Bottom Surgery  (gender-affirming genital surgery) | Personal | Positive |
|  |  |  |  | Negative |
|  |  |  |  | Neutral |
|  |  |  | Informational | |
|  |  |  | Other | |
|  |  | Facial Feminization Surgery | Personal | Positive |
|  |  |  |  | Negative |
|  |  |  |  | Neutral |
|  |  |  | Informational | |
|  |  |  | Other | |
|  |  | Brazilian Butt Lift | Personal | Positive |
|  |  |  |  | Negative |
|  |  |  |  | Neutral |
|  |  |  | Informational | |
|  |  |  | Other | |
|  |  | General Mentions | Personal | Positive |
|  |  |  |  | Negative |
|  |  |  |  | Neutral |
|  |  |  | Informational | |
|  |  |  | Other | |
|  |  | Other | Personal | Positive |
|  |  |  |  | Negative |
|  |  |  |  | Neutral |
|  |  |  | Informational | |
|  |  |  | Other | |
| **Mental Health** | Discussions about mental health diagnoses, treatments, and therapies. Includes personal experience and/or general information. | Medication, Treatment, and Therapy | Personal | Positive |
|  |  |  |  | Negative |
|  |  |  |  | Neutral |
|  |  |  | Informational | |
|  |  |  | Other | |
|  |  | Therapists/  Psychologists | Personal | Positive |
|  |  |  |  | Negative |
|  |  |  |  | Neutral |
|  |  |  | Informational | |
|  |  |  | Other | |
|  |  | Reparative/  Conversion Therapy | Personal | Positive |
|  |  |  |  | Negative |
|  |  |  |  | Neutral |
|  |  |  | Informational | |
|  |  |  | Other | |
|  |  | Suicidality | Personal | Positive |
|  |  |  |  | Negative |
|  |  |  |  | Neutral |
|  |  |  | Informational | |
|  |  |  | Other | |
|  |  | Eating Disorders | Personal | Positive |
|  |  |  |  | Negative |
|  |  |  |  | Neutral |
|  |  |  | Informational | |
|  |  |  | Other | |
|  |  | Substance Use Disorder/Addiction | Personal | Positive |
|  |  |  |  | Negative |
|  |  |  |  | Neutral |
|  |  |  | Informational | |
|  |  |  | Other | |
|  |  | Post Traumatic Stress Disorder | Personal | Positive |
|  |  |  |  | Negative |
|  |  |  |  | Neutral |
|  |  |  | Informational | |
|  |  |  | Other | |
|  |  | Other Diagnoses | Personal | Positive |
|  |  |  |  | Negative |
|  |  |  |  | Neutral |
|  |  |  | Informational | |
|  |  |  | Other | |
|  |  | General Mentions | Personal | Positive |
|  |  |  |  | Negative |
|  |  |  |  | Neutral |
|  |  |  | Informational | |
|  |  |  | Other | |
| **Menstruation** | Discussions about the management of periods and symptoms associated with menstruation. Includes personal experience and/or general information. |  | Personal | Positive |
|  |  |  |  | Negative |
|  |  |  |  | Neutral |
|  |  |  | Informational | |
|  |  |  | Other | |
| **Sexual Health** | Discussions about safe sex practices, sexual boundaries, sexual function, sexually transmitted infections (STIs), and consent. Includes personal experience and/or general information. |  | Personal | Positive |
|  |  |  |  | Negative |
|  |  |  |  | Neutral |
|  |  |  | Informational | |
|  |  |  | Other | |
| **Fertility and Family Planning** | Discussions about the ability to conceive or have biological children or alternative plans for creating a family, such as adoption or surrogacy. Includes personal experience and/or general information. |  | Personal | Positive |
|  |  |  |  | Negative |
|  |  |  |  | Neutral |
|  |  |  | Informational | |
|  |  |  | Other | |
| **Treatment Regrets** | Expressing regret about undergoing a certain treatment or regretting a certain aspect of that treatment. Includes personal experience and/or general information. |  | Personal | Positive |
|  |  |  |  | Negative |
|  |  |  |  | Neutral |
|  |  |  | Informational | |
|  |  |  | Other | |
| **General Mentions** | General mentions of medically transitioning and gender affirming healthcare, without naming a specific treatment. Includes personal experience and/or general information. |  | Personal | Positive |
|  |  |  |  | Negative |
|  |  |  |  | Neutral |
|  |  |  | Informational | |
|  |  |  | Other | |
| **Non-Medical Codes** | | | | |
| **Code** | **Subcode** | **Definition** | **Framework** | |
| **Non-Medical Affirmative Activities** | Changes to Appearance | Non-medical strategies to alter appearance to affirm gender identity. Includes practices such as binding, chest padding, packing, tucking, haircuts and styling, hair removal, make-up, clothing choices, and voice training. | Personal | |
|  |  |  | Informational | |
|  |  |  | Other | |
|  | Changes to Public Identity | Strategies to align outward or legally recognized identity with gender identity. Includes legal document changes, name and pronoun changes, and coming out. | Personal | |
|  |  |  | Informational | |
|  |  |  | Other | |
| **Social Experiences** | Interactions with Others Regarding Gender Identity | Interpersonal experiences in which one’s gender identity is affirmed or invalidated through comments, questions, or behaviors. | Personal | |
|  |  |  | Informational | |
|  |  |  | Other | |
|  | Bathroom Experiences | Experiences using public or shared bathrooms in the context of one’s gender identity. | Personal | |
|  |  |  | Informational | |
|  |  |  | Other | |
|  | Romantic Relationships | Discussions about romantic relationships or dating experiences in the context of one’s gender identity. | Personal | |
|  |  |  | Informational | |
|  |  |  | Other | |
| **Gender Identity Development** | Gender Discovery | Personal reflections and narratives about realizing and understanding one’s gender identity over time. | Personal | |
|  |  |  | Informational | |
|  |  |  | Other | |
|  | Social Support | Discussing support, protection, and encouragement one receives from their social network regarding their identity and wellbeing. | Personal | |
|  |  |  | Informational | |
|  |  |  | Other | |
|  | Resources | Providing recommendations for resources for education and gender identity development. | Personal | |
|  |  |  | Informational | |
|  |  |  | Other | |
|  | Explanations and Advice | Giving advice, providing definitions, and explaining concepts related to gender identity development. | Personal | |
|  |  |  | Informational | |
|  |  |  | Other | |
|  | Pride, Acceptance, and Empowerment | Expressing pride, acceptance and empowerment over one’s identity | Personal | |
|  |  |  | Informational | |
|  |  |  | Other | |
|  | Dysphoria | Discomfort and distress caused by having an incongruent assigned sex and gender identity. Includes discussions about activities, experiences, or personal attributes that cause gender dysphoria. | Personal | |
|  |  |  | Informational | |
|  |  |  | Other | |
|  | Passing | Discussions of being perceived by others in a way that is consistent (or inconsistent) with one’s gender identity. | Personal | |
|  |  |  | Informational | |
|  |  |  | Other | |
