# Supplementary material for: Medical Information Provided by Transgender and Gender-Diverse Content Creators on YouTube: Descriptive Content Analysis
Source: JMIR Form Res. 2025 Aug 29;9:e75787. doi: 10.2196/75787 (PMC12396793; doi:10.2196/75787)
Supplement: Multimedia Appendix 2 [file formative-v9-e75787-s002.docx]

| Coding Items | Observed Agreement | Prevalence-Adjusted Bias-Adjusted Kappa (PABAK) | Gwet's AC1 |
| --- | --- | --- | --- |
| All 47 items | 94% | 0.88 | 0.93 |
| Gender - Trans general | 90% | 0.80 | 0.83 |
| Gender - Trans man/FTM | 100% | 1.00 | 1.00 |
| Gender - Trans woman/MTF | 95% | 0.90 | 0.93 |
| Gender - Transmasc | 100% |  |  |
| Gender - Transfem | 100% |  |  |
| Gender - Non-binary | 95% | 0.90 | 0.94 |
| Gender - Gender non-conforming | 95% | 0.90 | 0.94 |
| Gender - Gender queer | 100% | 1.00 | 1.00 |
| Gender - Genderfluid | 100% |  |  |
| Gender - Cisgender | 100% |  |  |
| Gender - Additional | 100% |  |  |
| Puberty and androgen blockers | 100% | 1.00 | 1.00 |
| Hormone replacement therapy | 90% | 0.80 | 0.83 |
| Hormone replacement therapy - Estrogen | 95% | 0.90 | 0.94 |
| Hormone replacement therapy - Testosterone | 90% | 0.80 | 0.85 |
| Hormone replacement therapy - Not specified | 100% | 1.00 | 1.00 |
| Surgery | 90% | 0.80 | 0.81 |
| Surgery - Top surgery | 90% | 0.80 | 0.83 |
| Surgery - Bottom surgery | 85% | 0.70 | 0.83 |
| Surgery - Facial feminization surgery | 100% |  |  |
| Surgery - Brazilian butt lift | 100% |  |  |
| Surgery - General mentions | 100% |  |  |
| Surgery - Other | 100% |  |  |
| Mental health | 85% | 0.70 | 0.77 |
| Mental health - Medication, treatment, and therapy | 95% | 0.90 | 0.94 |
| Mental health - Therapists/psychologists | 100% |  |  |
| Mental health - Reparative/conversion therapy | 100% |  |  |
| Mental health - Suicidality | 100% |  |  |
| Mental health - Eating disorders | 100% |  |  |
| Mental health - Substance use disorder/addiction | 100% |  |  |
| Mental health - Depression | 95% | 0.90 | 0.94 |
| Mental health - Anxiety | 85% | 0.70 | 0.83 |
| Mental health - Post traumatic stress disorder | 100% |  |  |
| Mental health - Other diagnoses | 100% |  |  |
| Mental health - General mentions | 100% |  |  |
| Menstruation | 100% | 1.00 | 1.00 |
| Sexual health | 95% | 0.90 | 0.94 |
| Fertility and family planning | 95% | 0.90 | 0.92 |
| Treatment regrets | 100% |  |  |
| General mentions | 100% |  |  |
| No medical codes | 85% | 0.70 | 0.73 |
| Valence - Positive | 85% | 0.70 | 0.73 |
| Valence - Negative | 85% | 0.70 | 0.83 |
| Valence - Neutral | 85% | 0.70 | 0.79 |
| Framework - Personal | 95% | 0.90 | 0.90 |
| Framework - Information | 85% | 0.70 | 0.75 |
| Framework - Other | 70% | 0.40 | 0.42 |

**Appendix 2. Summary of interrater reliability statistics.**
